# Effect of Kramecyne on the Inflammatory Response in Lipopolysaccharide-Stimulated Peritoneal Macrophages

**DOI:** 10.1155/2013/762020

**Published:** 2013-03-13

**Authors:** E. Sánchez-Miranda, J. Lemus-Bautista, S. Pérez, J. Pérez-Ramos

**Affiliations:** Departamento de Sistemas Biológicos, Universidad Autónoma Metropolitana-Xochimilco, Calzada del Hueso 1100, Col. Villa Quietud, Coyoacán, 04960 México, DF, Mexico

## Abstract

Kramecyne is a new peroxide, it was isolated from *Krameria cytisoides*, methanol extract, and this plant was mostly found in North and South America. This compound showed potent anti-inflammatory activity; however, the mechanisms by which this compound exerts its anti-inflammatory effect are not well understood. In this study, we examined the effects of kramecyne on inflammatory responses in mouse lipopolysaccharide- (LPS-) induced peritoneal macrophages. Our findings indicate that kramecyne inhibits LPS-induced production of tumor necrosis factor (TNF-**α**) and interleukin- (IL-) 6. During the inflammatory process, levels of cyclooxygenase- (COX-) 2, nitric oxide synthase (iNOS), and nitric oxide (NO) increased in mouse peritoneal macrophages; however, kramecyne suppressed them significantly. These results provide novel insights into the anti-inflammatory actions and support its potential use in the treatment of inflammatory diseases.

## 1. Introduction

Inflammation is the physiological response of the body to stimuli, including infections and tissue injury [1]. However, excessive or prolonged inflammation can prove harmful, contributing to the pathogenesis of various diseases, including arthritis, asthma, multiple sclerosis, inflammatory bowel disease, and atherosclerosis [2, 3]. Macrophages play critical roles in the inflammation process.

The LPS-induced inflammation was first understood at the second half of the 1980s by Stuehr and Marletta [4]. The macrophages are activated by an LPS-induced inflammatory response caused by the release of several inflammatory mediators including nitric oxide (NO), cyclooxygenase- (COX-) 2, interleukin- (IL-) 6, and tumor necrosis factor- (TNF-) *α* [5, 6].

Therefore, the effect of compounds that prevent inflammation can be evaluated by monitoring the production of TNF-*α*, IL-6, and/or NO. The regulation of these mediators is important for understanding the inflammatory process and because they serve as a potential site for intervention in inflammatory diseases [7]. Currently inflammatory disorders are treated with corticosteroids, nonsteroidal (NSAIDs), and biologics drugs. However, all of these drugs trigger adverse side effects [8]. For these reasons, researchers on new compounds with anti-inflammatory activity and natural products are an important source with fewer adverse effects. Recently the novel compound kramecyne was isolated from *Krameria cytisoides* and showed anti-inflammatory activities in ear edema induced by 12-O-tetradecanoylphorbol-13-acetate (TPA) and paw edema induced by carrageenan [9].

Hence, the aim of this study wasusingmacrophages activated with LPS to know the effect of kramecyne on the expressions of TNF-*α*, NO, iNOS, COX-2, and IL-6 genes in order to identify the mechanism of action of this natural anti-inflammatory product.

## 2. Materials and Methods

### 2.1. Plant Material

*K. cytisoides* was collected in Las Comadres Municipality of Guadalcazar, San Luis Potosi State, Mexico. The identification of the plant was confirmed by an expert taxonomist. A voucher specimen (SPLM44560) was deposited into the Isidro Palacios Herbarium of the Universidad Autónoma de San Luis Potosí.

### 2.2. Isolation of Kramecyne

Shade-dried leaves of *K. cytisoides* were reduced to powder. A portion (200 g) was defatted with hexane (2 L) under reflux for 4 h and then extracted into MeOH (2 L) under reflux for 4 h. The methanol extract was concentrated to half the original volume under reduced pressure, and a dark brown solid was obtained with 3% yield (m.p. 172°C, dec.). The compound purity was determined by thin-layer chromatography. The compound was identified as kramecyne with FTIR *ν* cm^−1^ (solid) 3332 (O–H), 2919 and 2486 (C–H), 1608 (skeleton), 1443 (C–C), 1284, 1108 (C–O), and 800 (O–O) ^1^H-NMR (500 MHz, methanol-d_4_): *δ* 3.61, d(11.47), 3.63, d(11.47), 3.628, d(11.50), 3.668, d(11.50), 3.69, m, 3.74, dd (3.19). ^13^C-NMR (500 MHz, methanol-d_4_): *δδ* 62.12 (CH_2_), 62.85 (CH_2_), 63.40 (CH_2_), 75.15 (C), 73.17 (CH) [9].

### 2.3. Cell Culture

One and a half milliliters of thioglycolate medium (4%) was injected into the peritoneal cavity of BALB/c mice. After 72 h, macrophages were collected by peritoneal lavage with 10 mL cold PBS buffer. The buffer was centrifuged to isolate the cells. Cells were counted in a Neubawer chamber, plated in 12-well plates, and cultured for 24 h. The nonadherent cells were removed, and adherents cells were cultured with fresh medium. Peritoneal macrophages were cultured with RPMI supplemented with 10% inactivated fetal bovine serum (FBS), penicillin (100 units/mL), and streptomycin (100 *μ*g/mL) under CO_2_ (5%) at 37°C.

### 2.4. Cell Viability Using Violet Crystal Exclusion Assay

Peritoneal macrophages (1 × 10^6^ cells/well) were cultured in a 12-well plate for 24 h in under CO_2_ (5%) at 37°C. The medium was removed and replaced with fresh medium containing kramecyne alone and kramecyne plus LPS at varying concentrations (31.25, 62.5, 125, and 250 *μ*g/mL) and then incubated for 24 h. Cell viability was assessed by adding 200 *μ*L of 0.4% crystal violet solution followed by incubation for 30 min. At room temperature. After the crystal violet solution was replaced by acetic acid (33%), it was recovered and its absorbance was measured at 540 nm in a microplate reader [10].

### 2.5. Determination of Nitric Oxide Production

Nitrite production was measured by the Griess reaction [11]. Peritoneal macrophages (1 × 10^6^ cells/well) were incubated overnight. After the medium was removed and replaced with fresh medium containing kramecyne alone and kramecyne plus LPS varying concentrations (31.25, 62.5, 125, and 250 *μ*g/mL), macrophages were incubated for 2 h. Thereafter, lipopolysaccharide (LPS, *Escherichia coli* O111:B4, 1 *μ*g/mL) was added followed by 24 h incubation. The supernatant was collected. One hundred microliters of supernatant was treated with 100 *μ*L of Griess reagent (1% sulphanilamide, 0.1% naphtylethylenediamine dihydrochloride, and 5% orthophosphoric acid), and the mixture was incubated at room temperature for 5 min. Then the absorbance was measured at 540 nm in a microplate reader. The amount of nitrite in the sample was determined using sodium nitrite for the standard curve.

### 2.6. RT-PCR Analysis of mRNA

Peritoneal macrophages (2 × 10^6^ cells/well) were cultured kramecyne alone and kramecyne and LPS, varying concentrations of kramecyne (31.25, 62.5, 125, and 250 *μ*g/mL) in 12-well plates for 2 h, stimulated with LPS (1 *μ*g/mL), and incubated for 24 h. The inhibitory effect of kramecyne on mRNA expression of proinflammatory cytokines (IL-6 and TNF-*α*) and mediators (iNOS and COX-2) was determined by semiquantitative RT-PCR. The PCR products were normalized to the amount of 18S ribosomal RNA. Primers were designed using Primer BLAST (http://www.ncbi.nlm.nih.gov/tools/primer-blast/) (Table 1).

### 2.7. Measurement of the Production of Proinflammatory Cytokines (TNF-*α* and IL-6)

Peritoneal macrophages were cultured with varying concentration of kramecyne (31.25, 62.5, 125, and 250 *μ*g/mL) in 12-well culture plates for 2 h, stimulated with LPS (1 *μ*g/mL), and incubated for 24 h. The inhibitory effect of kramecyne on the production of proinflammatory cytokines (IL-6 and TNF-*α*) was determined by examining the collected supernatants. Cytokines concentration was measured using a mouse ELISA kit (eBioscience).

### 2.8. Statistical Analysis

All values were expressed as the mean ± SEM. The differences between mean values of normally distributed data were assessed with a one-way ANOVA (Newman Keuls *t*-test). Statistical significance was accepted at *P* < 0.05.

## 3. Results

### 3.1. Effect of Kramecyne on Cell Viability

**Figure 1:**
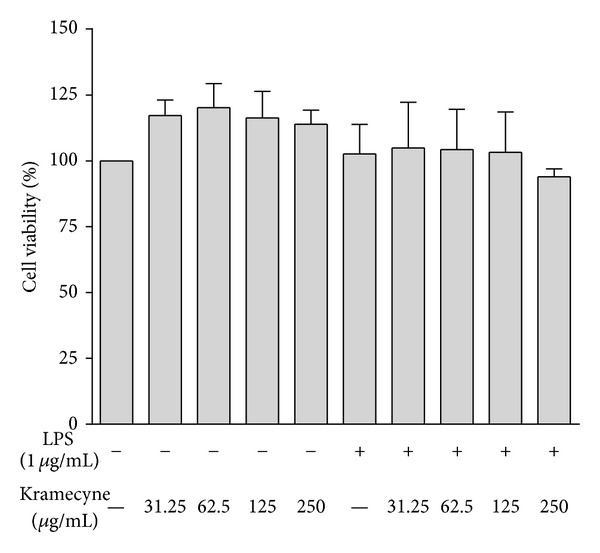
Effect of kramecyne on cell viability in peritoneal macrophages. Peritoneal macrophages were treated with kramecyne at 31.25, 62.5, 125, and 250 *μ*g/mL in the presence or absence of 1 *μ*g/ml LPS for 24 hrs. Cell viability was examined with violet crystal. Results are expressed as the percentage of surviving cells relative to control cells. Date represent mean ± SEM of three independent experiments; each was performed in triplicate.

The effect of kramecyne and kramecyne plus LPS on the cell viability of peritoneal macrophages is shown in Figure 1. These compounds did not demonstrate any toxicity at the conditions tested (31.25, 62.5, 125, and 250 *μ*g/mL).

### 3.2. Inhibitory Effect of Kramecyne in the NO Production in Peritoneal Macrophages

**Figure 2:**
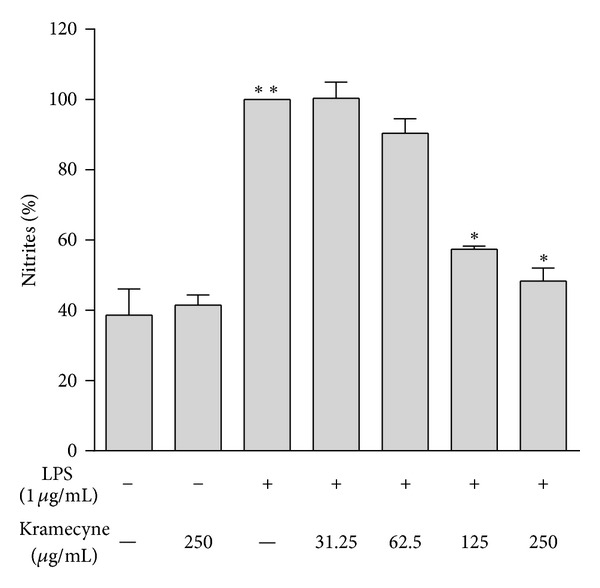
Effect of kramecyne on NO production in peritoneal macrophages LPS stimulated. The Griess reagent assay was carried out to measure nitrite produced as described in the materials and methods. Date represent mean ± SEM of three independent experiments performed in triplicate; **P < 0.05 versus basal and kramecyne group; *P < 0.05 versus LPS group.

We found that macrophages produced considerable amount of NO under basal conditions. After stimulation with LPS, NO production was increased significantly. However, when kramecyne was added at 31.25, 62.5, 125, and 250 *μ*g/mL, the NO levels were diminished by 5.3, 11.0, 37.0, and 47.3%, respectively (Figure 2). No significant difference in NO levels was found between cells under basal conditions and those treated with 125 or 250 *μ*g/mL of compound. 

### 3.3. Inhibitory Effect of Kramecyne on iNOS, COX-2, TNF-*α*, and IL-6 mRNA Expression in LPS-Stimulated Peritoneal Macrophages

**Figure 3:**
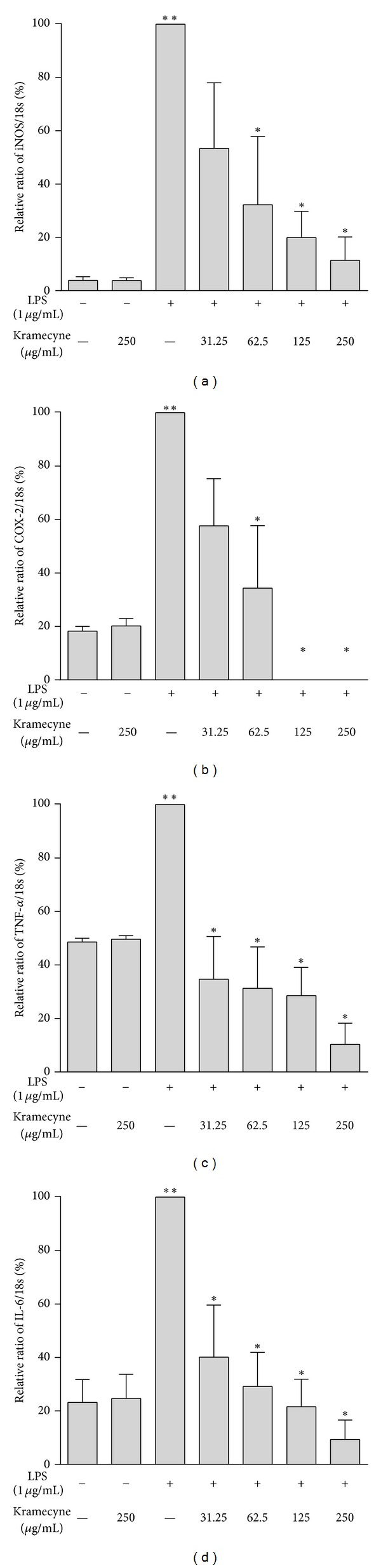
Effect of kramecyne on LPS-induced mRNA expression of (a) iNOS, (b) COX-2, (c) TNF-*α*, and (d) IL-6 in peritoneal macrophages. Total RNA (1 *μ*g) was prepared and analyzed by RT-PCR, as described in the material and methods. Date represent mean ± SEM of three independent experiments; **P < 0.05 versus basal and kramecyne group; *P < 0.05 versus LPS group.

The expression of iNOS and COX-2 mRNAs was increased in LPS-stimulated macrophages. Kramecyne inhibited iNOS production in a concentration-dependent manner. At 31.25, 62.5, 125, and 250 *μ*g/mL, kramecyne inhibited iNOS significantly (53.4, 68.0, 80.0, and 88.7%, resp.) (Figure 3(a)). At concentration of 125 and 250 *μ*g/mL, kramecyne completely suppressed mRNA expression of COX-2 mRNA (Figure 3(b)).

Kramecyne also significantly inhibited the expression of TNF-*α* and IL-6 in LPS-stimulated macrophages, and the effect was concentration dependent (Figure 3(c)). TNF-*α* expression diminished by 65.0, 68.7, 71.5, and 90.0% at 31.25, 62.5, 125, and 250 *μ*g/mL, respectively. In the case of IL-6, kramecyne suppressed its expression by 60.0, 70.8, 78.0, and 90.7% (Figure 3(d)).

### 3.4. Inhibitory Effect of Kramecyne on TNF-*α* and IL-6 Production in LPS-Stimulated Peritoneal Macrophages

**Figure 4:**
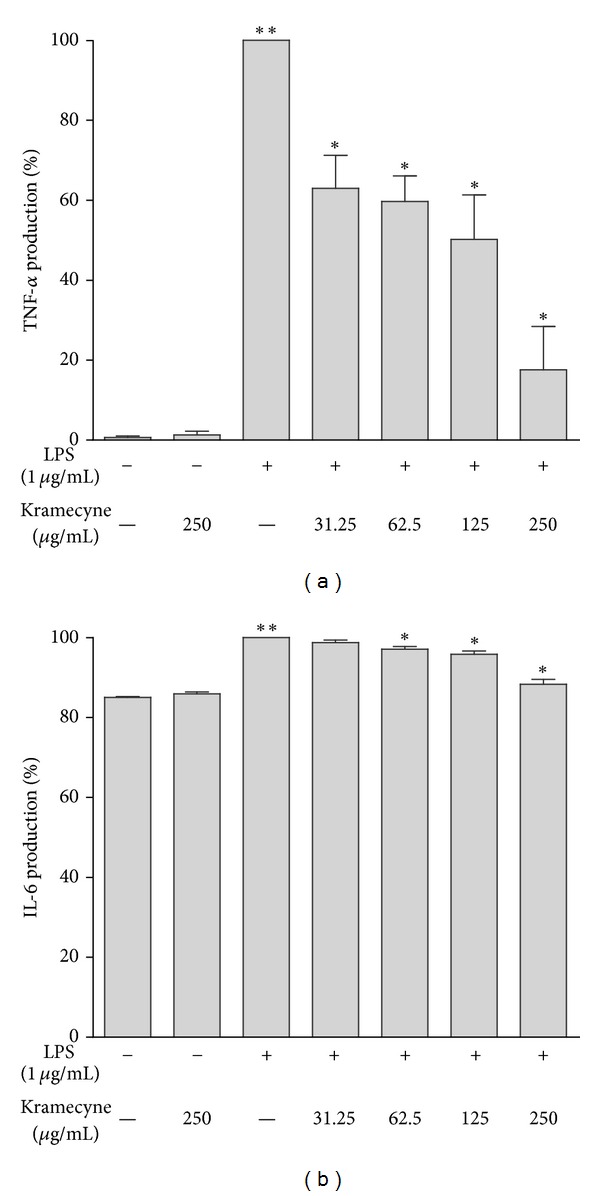
Inhibitory effect of kramecyne on the (a) TNF-*α* and (b) IL-6 cytokines production in peritoneal macrophages. Concentration in the supernatants was determined by ELISA. The results are the mean values ± SEM for five independent experiments; **P < 0.05 versus basal and kramecyne group; *P < 0.05 versus LPS group.

Kramecyne significantly reduced TNF-*α* and IL-6 production in LPS-activated macrophages, and the responses were dose dependent (Figures 4(a) and 4(b)). TNF-*α* was significantly inhibited by kramecyne at 31.25, 62.5, 125, and 250 *μ*g/mL (37.3, 40.4, 48.0, and 81.0%, resp.). At 250 *μ*g/mL, kramecyne completely suppressed IL-6 production as compared to macrophages under basal condition. 

## 4. Discussion

Inflammation is the first response of the immune system to infection or irritation, and macrophages play a crucial role during the inflammatory process [12]. Lipopolysaccharide (LPS) is an endotoxin, an integral outer membrane component of Gram-negative bacteria, and triggers the most potent microbial initiators of inflammatory response, including septic shock, fever, and microbial invasion [13]. Murine and human macrophages exhibit a particularly vigorous response to LPS, which induces a variety of inflammatory modulators such as NO, TNF-*α*, IL-6, and PGs [14]. These proinflammatory mediators are regarded as essential anti-inflammatory targets [15, 16]. For this reason, the stimulation macrophages with LPS constitute an excellent model for the screening and subsequent evaluation of the effects of candidate drugs on the inflammatory pathway. The excessive or prolonged inflammation can prove harmful, contributing to the pathogenesis of a variety of diseases, including arthritis, asthma, multiple sclerosis, inflammatory bowel disease, and atherosclerosis [17–19]. Therefore, agents that regulate cytokines and inflammatory mediators may have therapeutic effects. Various *in vivo* and *in vitro* experimental models have been set up to assess inhibitory effect of various natural products on these inflammatory mediators [20–25].

NO is an intracellular messenger which regulates vascular relaxation and participates in the process of elimination of pathogens and tumour cells, and it is involved in promoting inflammatory responses [26–28]. Evidence indicates that excessive production of NO resulted in excess inflammatory reaction deleterious to the human body in the inflammation process [26, 29–31]. NO by macrophages may lead to various pathological disorders such as inflammation acute and chronic [32], carcinogenicity, cytotoxicity, and autoimmune diseases [33]. The free radical nature on NO and its high reactivity allow NO to react with oxygen to produce peroxynitrite (ONOO^−^), which make it a potent pro-oxidant molecule that is able to induce oxidative damage, and can be potentially harmful towards cellular targets [34]. Therefore, NO production can be used as a measure of the progression of inflammation and inhibition of NO might have potential therapeutic value when related to inflammation-associated disease [35]. NO is produced in physiological and pathophysiological conditions by three distinct isoforms of nitric oxide synthase (NOS): endothelial NOS (eNOS), neuronal NOS (nNOS), and inducible NOS (iNOS) [36, 37], while eNOS and nNOS are constitutively expressed and regulated by Ca^2+^-calmodulin [38]. The activity of iNOS is regulated at the transcription levels by mediators such as IL-1, IL-6, INF-*γ*, and TNF-*α* [39, 40]. Although TNF-*α* is not an inducer of iNOS, it is crucial for synergistic induction of NO synthesis in INF-*γ* and/or LPS-stimulated murine peritoneal macrophages and regulates NO synthesis *in vivo* [41]. Therefore, a direct and/or indirect modulation of macrophage-mediated NO production may reduce these inflammatory diseases. In the present study, we examined the mechanism of action of kramecyne and found that this compound decreased NO production and iNOS expression in LPS-stimulated macrophages peritoneal macrophages, and this effect was independent of concentration (Figure 2).

Cyclooxygenase-2 (COX-2) is expressed in response to inflammatory and other physiological stimuli and growth factors. It is involved in the synthesis of prostaglandins (PG) that mediated temperature and pain and supported the inflammation process [42, 43]. COX-2 is found in high concentrations in patients with inflammatory diseases, and the levels of PGs are increased if inflammation continues [44, 45]. The inhibition of COX-2 is clinically relevant because PG production is thought to be responsible for the antipyretic, anti-inflammatory, and analgesic proprieties of NSAIDs [46]. NSAIDs inhibit both isoforms of COX, and their adverse effects, mainly gastrointestinal ulcers, are attributed to the inhibition of the release of gastroprotective prostaglandins produced via COX-1 pathways [47, 48]. Many studies have demonstrated that compounds that selectively inhibit COX-2 cause less damage to gastric mucosa [8]. Our results showed that kramecyne significantly attenuated COX-2 mRNA expression at 250 *μ*g/mL in LPS-stimulated macrophages. Therefore, it seems quite reasonable to propose that kramecyne inhibits PGE2s production. Moreover, further studies are required to determine whether kramecyne is a selective inhibitor of COX-2.

Inflammatory disorders are characterized by the production of a significant amount of cytokines such as TNF-*α*, IL-6, and IL-1*β* [49]. TNF-*α* is a potent proinflammatory cytokine, commonly released by macrophages. It upregulated other proinflammatory cytokines like IL-6 that is responsible for the induction and perpetuation of inflammation [50]. These cytokines may cause severe tissue damage, septic shock, atherosclerosis, cytotoxicity, and rheumatoid arthritis [51, 52]. These two cytokines are known to act as proinflammatory mediators *in vitro* and *in vivo*.

IL-6 is a multifunctional cytokine with pro- and anti-inflammatory properties that plays a central role in the regulation of defense mechanisms, haematopoiesis, and the production of acute phase proteins [53]. In addition, the overexpression of IL-6 is involved in physiological conditions such as rheumatoid arthritis [54]. In this study, we found that kramecyne reduced TNF-*α* and IL-6 mRNA expressions and secretion, in LPS-stimulated macrophages, in a concentration-dependent manner (Figure 3).

Previous studies have demonstrated that the expression of iNOS is stimulated by proinflammatory cytokines, including IL-1*β* and TNF-*α*, which contribute to tissue damage and multiple organ failure [55]. Additionally, Schrader et al. [56] suggested that TNF-*α* stimulates IL-6 production and that this is a prerequisite for increased NO production. These results suggest that the inhibition of iNOS/NO by kramecyne may be associated with the attenuation of TNF-*α* and IL-6 production.

Many studies have demonstrated that the expression of enzymes and cytokines proinflammatory are largely regulated by transcription activation. Nuclear factor kappa B (NF*κ*B) is essential for the transcription of genes that encode for inflammatory molecules which participate in the acute inflammatory responses, including iNOS, COX-2, TNF-*α*, and IL-6 in the monocyte-macrophage lineage, and this transcription factor is activated by LPS [57]. However, this study is limited to understand the effect of kramecyne on gene expression of iNOS, COX-2, TNF-*α*, and IL-6 and the production of NO. Therefore, it will be interesting to understand the effect of kramecyne at transcriptional activation level involving NF*κ*B protein. This transcription factor plays a crucial role in the regulation of cellular responses, and the mitigation of this factor is considered a good therapeutic option for inflammation. Our results demonstrated that kramecyne inhibited the mediators regulated for NF*κ*B, and this fact might suggest that this compound acts on the transcription factor, but other studies are required to confirm this proposal.

Kramecyne showed anti-inflammatory activities in TPA-induced mouse ear edema and carrageenan-induced rat paw edema. It has been observed that kramecyne does not exhibit toxic effects even at a dose of 5000 mg/kg in a model of acute toxicity [9]. Taken together with the cells viability studies, these results support the low toxicity of kramecyne and encourage the possibility of using it as anti-inflammatory.

## 5. Conclusion

Kramecyne is a potent inhibitor of iNOS, COX-2, NO, TNF-*α*, and IL-6 production at the transcriptional level in LPS-stimulated macrophages. The mechanism of inhibition for NO production appears to be due to a downregulation of iNOS mRNA expression, which might be associated with the attenuation of TNF-*α* and IL-6 production. Although the exact mechanisms for the anti-inflammatory activity of kramecyne are not fully known, these findings suggest that kramecyne may be as potent compound for the treatment of inflammatory diseases.

## Figures and Tables

**Table 1:** List of sequences used for RT-PCR.

Gene	Sequence	Length (bP)
iNOS	Forward: ACCTTGGAGTTCACCCAGT	170
	Reverse: ACCACTCGTACTTGGGATGC	
COX-2	Forward: GCGAGCTAAGAGCTTCAGGA	212
	Reverse: TCATACATTCCCCACGGTTT	
TNF-*α*	Forward: CTGGGACAGTGACCTGGACT	204
	Reverse: GCACCTCAGGGAAGAGTCTG	
IL-6	Forward: AGTTGCCTTCTTGGGACTGA	159
	Reverse: TCCACGATTTCCCAGAGAAC	
18s	Forward: GTAACCCGTTGAACCCCATT	140
	Reverse: CCATCCAATCGGTAGTAGCG	

## References

[1] Dung NT, Bajpai VK, Yoon JI, Kang SC (2009). Anti-inflammatory effects of essential oil isolated from the buds of *Cleistocalyx operculatus* (Roxb.) Merr and Perry. *Food and Chemical Toxicology*.

[2] Nathan C (2002). Points of control in inflammation. *Nature*.

[3] Rankin JA (2004). Biological mediators of acute inflammation. *Advanced Critical Care*.

[4] Stuehr DJ, Marletta MA (1985). Mammalian nitrate biosynthesis: mouse macrophages produce nitrite and nitrate in response to *Escherichia coli* lipopolysaccharide. *Proceedings of the National Academy of Sciences of the United States of America*.

[5] Kanno SI, Shouji A, Tomizawa A (2006). Inhibitory effect of naringin on lipopolysaccharide (LPS)-induced endotoxin shock in mice and nitric oxide production in RAW 264.7 macrophages. *Life Sciences*.

[6] Lee MY, Lee JA, Seo CS (2011). Anti-inflammatory activity of *Angelica dahurica* ethanolic extract on RAW264.7 cells via upregulation of heme oxygenase-1. *Food and Chemical Toxicology*.

[7] Davis KL, Martin E, Turko IV, Murad F (2001). Novel effects of nitric oxide. *Annual Review of Pharmacology and Toxicology*.

[8] Gautam R, Jachak SM (2009). Recent developments in anti-inflammatory natural products. *Medicinal Research Reviews*.

[9] Pérez S, Sánchez E, Martínez M, Zavala MA, Pérez C (2012). Kramecyne—a new anti-inflammatory compound isolated from *Krameria cytisoides*. *Molecules*.

[10] Elmann A, Mordechay S, Erlank H, Telerman A, Rindner M, Ofir R (2011). Anti-Neuroinflammatory effects of the extract of *Achillea fragrantissima*. *BMC Complementary and Alternative Medicine*.

[11] Ahn KS, Noh EJ, Zhao HL, Jung SH, Kang SS, Kim YS (2005). Inhibition of inducible nitric oxide synthase and cyclooxygenase II by *Platycodon grandiflorum* saponins via suppression of nuclear factor-*κ*B activation in RAW 264.7 cells. *Life Sciences*.

[12] Ialenti A, Moncada S, Di Rosa M (1993). Modulation of adjuvant arthritis by endogenous nitric oxide. *British Journal of Pharmacology*.

[13] Dobrovolskaia MA, Vogel SN (2002). Toll receptors, CD14, and macrophage activation and deactivation by LPS. *Microbes and Infection*.

[14] Adams DO, Hamilton TA (1984). The cell biology of macrophage activation. *Annual Review of Immunology*.

[15] Larsen GL, Henson PM (1983). Mediators of inflammation. *Annual Review of Immunology*.

[16] Lawrence T, Willoughby DA, Gilroy DW (2002). Anti-inflammatory lipid mediators and insights into the resolution of inflammation. *Nature Reviews Immunology*.

[17] Guzik TJ, Korbut R, Adamek-Guzik T (2003). Nitric oxide and superoxide in inflammation and immune regulation. *Journal of Physiology and Pharmacology*.

[18] Nathan C (2002). Points of control in inflammation. *Nature*.

[19] Rankin JA (2004). Biological mediators of acute inflammation. *AACN Clinical Issues*.

[20] Kazłowska K, Hsu T, Hou CC, Yang WC, Tsai GJ (2010). Anti-inflammatory properties of phenolic compounds and crude extract from *Porphyra dentata*. *Journal of Ethnopharmacology*.

[21] Michelini FM, Ramírez JA, Berra A, Galagovsky LR, Alché LE (2008). Anti-herpetic and anti-inflammatory activities of two new synthetic 22,23-dihydroxylated stigmastane derivatives. *Journal of Steroid Biochemistry and Molecular Biology*.

[22] Mueller M, Hobiger S, Jungbauer A (2010). Anti-inflammatory activity of extracts from fruits, herbs and spices. *Food Chemistry*.

[23] Paulino N, Rodrigues NC, Pardi PC (2009). Evaluation of anti-inflammatory effect of synthetic 1,5-bis(4-acetoxy-3-methoxyphenyl)-1,4-pentadien-3-one, HB2. *Bioorganic and Medicinal Chemistry*.

[24] Prawan A, Saw CLL, Khor TO (2009). Anti-NF-*κ*B and anti-inflammatory activities of synthetic isothiocyanates: effect of chemical structures and cellular signaling. *Chemico-Biological Interactions*.

[25] Van Q, Nayak BN, Reimer M, Jones PJH, Fulcher RG, Rempel CB (2009). Anti-inflammatory effect of Inonotus obliquus, *Polygala senega* L., and *Viburnum trilobum* in a cell screening assay. *Journal of Ethnopharmacology*.

[26] Li CQ, He LC, Jin JQ (2007). Atractylenolide I and atractylenolide III inhibit lipopolysaccharide- induced TNF-*α* and NO production in macrophages. *Phytotherapy Research*.

[27] Ponnuchamy B, Khalil RA (2009). Cellular mediators of renal vascular dysfunction in hypertension. *American Journal of Physiology*.

[28] Zulkhurnain U, Mohamed IAM, Mohd IA, Mohd FAJ, Tan ML (2011). Mitragynine inhibits the COX-2 mRNA expression and prostaglandin E2 production induced by lipopolysaccharide in RAW 264.7 macrophage cells. *Journal of Ethnopharmacology*.

[29] Ialenti A, Ianaro A, Moncada S, Di Rosa M (1992). Modulation of acute inflammation by endogenous nitric oxide. *European Journal of Pharmacology*.

[30] Iuvone T, Carnuccio R, Di Rosa M (1994). Modulation of granuloma formation by endogenous nitric oxide. *European Journal of Pharmacology*.

[31] Klosterhalfen B, Bhardwaj RS (1998). Septic shock. *General Pharmacology*.

[32] Clancy RM, Abramson SB (1995). Nitric oxide: a novel mediator of inflammation. *Proceedings of the Society for Experimental Biology and Medicine*.

[33] Sarkar D, Saha P, Gamre S (2008). Anti-inflammatory effect of allylpyrocatechol in LPS-induced macrophages is mediated by suppression of iNOS and COX-2 via the NF-*κ*B pathway. *International Immunopharmacology*.

[34] Epe B, Ballmaier D, Roussyn I, Briviba K, Sies H (1996). DNA damage by peroxynitrite characterized with DNA repair enzymes. *Nucleic Acids Research*.

[35] Nurul Islam Md, Joo Choi R, Eun Jin S (2012). Mechanism of anti-inflammatory activity of umbelliferone 6-carboxylic acid isolated from *Angelica decursiva*. *Journal of Ethnopharmacology*.

[36] Marletta MA (1993). Nitric oxide synthase structure and mechanism. *Journal of Biological Chemistry*.

[37] Nathan C, Xie QW (1993). Regulation of biosynthesis of nitric oxide. *Journal of Biological Chemistry*.

[38] Alderton WK, Cooper CE, Knowles RG (2001). Nitric oxide synthases: structure, function and inhibition. *Biochemical Journal*.

[39] Sung CS, Wong CS (2007). Cellular mechanisms of neuroinflammatory pain: the role of interleukin-1*β*. *Acta Anaesthesiologica Taiwanica*.

[40] Vuolteenaho K, Moilanen T, Knowles RG, Moilanen E (2007). The role of nitric oxide in osteoarthritis. *Scandinavian Journal of Rheumatology*.

[41] Harbrecht BG, Di Silvio M, Demetris AJ, Simmons RL, Billiar TR (1994). Tumor necrosis factor-*α* regulates in vivo nitric oxide synthesis and induces liver injury during endotoxemia. *Hepatology*.

[42] Tsatsanis C, Androulidaki A, Venihaki M, Margioris AN (2006). Signalling networks regulating cyclooxygenase-2. *International Journal of Biochemistry and Cell Biology*.

[43] Aronoff D, Neilso E (2001). Antipyretics: mechanism of action and clinical use in fever suppression. *American Journal of Medicine*.

[44] Masferrer JL, Zweifel BS, Manning PT (1994). Selective inhibition of inducible cyclooxygenase 2 *in vivo* is anti inflammatory and nonulcerogenic. *Proceedings of the National Academy of Sciences of the United States of America*.

[45] Van Q, Nayak BN, Reimer M, Jones PJH, Fulcher RG, Rempel CB (2009). Anti-inflammatory effect of *Inonotus obliquus*, *Polygala senega* L., and *Viburnum trilobum* in a cell screening assay. *Journal of Ethnopharmacology*.

[46] Clàiria J (2003). Cyclooxygenase-2 biology. *Current Pharmaceutical Design*.

[47] Allison MC, Howatson AG, Torrance CJ, Lee FD, Russell RI (1992). Gastrointestinal damage associated with the use of nonsteroidal antiinflammatory drugs. *The New England Journal of Medicine*.

[48] Eberhart CE, Dubois RN (1995). Eicosanoids and the gastrointestinal tract. *Gastroenterology*.

[49] Holtmann H, Resch K (1995). Cytokine. *Die Naturwissenchaften*.

[50] Beutler B, Cerami A (1989). The biology of cachectin/TNF-*α* primary mediator of the host response. *Annual Review of Immunology*.

[51] Hseu YC, Wu FY, Wu JJ (2005). Anti-inflammatory potential of *Antrodia Camphorata* through inhibition of iNOS, COX-2 and cytokines via the NF-*κ*B pathway. *International Immunopharmacology*.

[52] Garlanda C, Di Liberto D, Vecchi A (2007). Damping excessive inflammation and tissue damage in *Mycobacterium tuberculosis* infection by toll IL-1 receptor 8/single Ig IL-1-related receptor, a negative regulator of IL-1/TLR signaling. *Journal of Immunology*.

[53] Park SJ, Jung JA, Lee SJ, Kim HM (1999). The phosphatidylinositol 3 kinase inhibitor, LY294002, block TNF-*α* and IL-6 production in activated RAW-264. 7 cells. *Bulletin of Life Science and Biotechnology*.

[54] Van Q, Nayak BN, Reimer M, Jones PJH, Fulcher RG, Rempel CB (2009). Anti-inflammatory effect of *Inonotus obliquus*, *Polygala senega* L., and *Viburnum trilobum* in a cell screening assay. *Journal of Ethnopharmacology*.

[55] Marcus JS, Karackattu SL, Fleegal MA, Sumners C (2003). Cytokine-stimulated inducible nitric oxide synthase expression in astroglia: role of Erk mitogen-activated protein kinase and NF-*κ*B. *GLIA*.

[56] Schrader LI, Kinzenbaw DA, Johnson AW, Faraci FM, Didion SP (2007). IL-6 deficiency protects against angiotensin II-induced endothelial dysfunction and hypertrophy. *Arteriosclerosis, Thrombosis, and Vascular Biology*.

[57] Barnes PJ, Karin M (1997). Nuclear factor-*κ*B—a pivotal transcription factor in chronic inflammatory diseases. *The New England Journal of Medicine*.

